# Supplementary material for: Dynamics of Photoinduced Charge Carriers in Metal-Halide Perovskites
Source: Nanomaterials (Basel). 2024 Oct 30;14(21):1742. doi: 10.3390/nano14211742 (PMC11547212; doi:10.3390/nano14211742)
Supplement: Supplementary file 1 [file nanomaterials-14-01742-s001.zip › SupMat_muPCD_perovskites_1stround_resubmit.pdf]

Supplementary Materials: Dynamics of Photoinduced Charge Carriers in Metal-Halide Perovskites

András Bojtor[1,2], Dávid Krisztián[1,2], Ferenc Korsós[2], Sándor Kollarics[1,3], Gábor Paráda[2], Márton Kollár[4], Endre Horváth[4], Xavier Mettan[4], Bence G. Márkus[3,5,6], László Forró[5] and Ferenc Simon[1,3,5,7]*

1. Shockley-Read-Hall recombination for trap states with both charge types

As mentioned in the main text, the rate equations for the Shockley-Read-Hall [1–3] mechanism were given for a trap state which is assumed to be either neutral of negatively charged. The symmetry between negative and positive charge carrier dictates that trap states are possible which can be neutral or positively charged. Then, the full set of a four-level rate equations is obtained as:

$$
\begin{aligned}
\frac{\mathrm{d}n}{\mathrm{d}t} &= \overbrace{-C_{\text{e,n-SRH}}\left(N_{\text{n-SRH}} - n_{\text{n-SRH}}\right)n + E_{\text{e,n-SRH}}n_{\text{n-SRH}}}^{\text{CB}\longleftrightarrow\text{n-SRH}} \\
&\quad \overbrace{-C_{\text{e,p-SRH}}p_{\text{p-SRH}}n + E_{\text{e,p-SRH}}\left(N_{\text{p-SRH}} - p_{\text{p-SRH}}\right)}^{\text{CB}\longleftrightarrow\text{p-SRH}}, \\
\frac{\mathrm{d}n_{\text{n-SRH}}}{\mathrm{d}t} &= \overbrace{+C_{\text{e,n-SRH}}\left(N_{\text{n-SRH}} - n_{\text{n-SRH}}\right)n - E_{\text{e,n-SRH}}n_{\text{n-SRH}}}^{\text{CB}\longleftrightarrow\text{n-SRH}} \\
&\quad \overbrace{-C_{\text{h,n-SRH}}n_{\text{n-SRH}}p + E_{\text{h,n-SRH}}\left(N_{\text{n-SRH}} - n_{\text{n-SRH}}\right)}^{\text{VB}\longleftrightarrow\text{n-SRH}}, \\
\frac{\mathrm{d}p_{\text{p-SRH}}}{\mathrm{d}t} &= \overbrace{-C_{\text{e,p-SRH}}p_{\text{p-SRH}}n + E_{\text{e,p-SRH}}\left(N_{\text{p-SRH}} - p_{\text{p-SRH}}\right)}^{\text{CB}\longleftrightarrow\text{p-SRH}} \\
&\quad \overbrace{+C_{\text{h,p-SRH}}\left(N_{\text{p-SRH}} - p_{\text{p-SRH}}\right)p - E_{\text{h,p-SRH}}p_{\text{p-SRH}}}^{\text{VB}\longleftrightarrow\text{p-SRH}}, \\
\frac{\mathrm{d}p}{\mathrm{d}t} &= \overbrace{-C_{\text{h,n-SRH}}n_{\text{n-SRH}}p + E_{\text{h,n-SRH}}\left(N_{\text{n-SRH}} - n_{\text{n-SRH}}\right)}^{\text{VB}\longleftrightarrow\text{n-SRH}} \\
&\quad \overbrace{-C_{\text{h,p-SRH}}\left(N_{\text{p-SRH}} - p_{\text{p-SRH}}\right)p + E_{\text{h,p-SRH}}p_{\text{p-SRH}}}^{\text{VB}\longleftrightarrow\text{p-SRH}},
\end{aligned}
\tag{1}
$$

where $p_{\text{p-SRH}}$ denotes the (positive) charge-carrier density of the trap level which can be positively charged. $N_{\text{n-SRH}}$ and $N_{\text{p-SRH}}$ denotes the density of the two types of trap levels. The meaning of the transition processes:
$C_{\text{e,n-SRH}}$: an electron is captured into the negative SRH center. It is possible if it is originally charge neutral, i.e. unoccupied with population $\left(N_{\text{n-SRH}} - n_{\text{n-SRH}}\right)$.
$E_{\text{e,n-SRH}}$: an electron is emitted from the negative SRH center. It is possible if the center is negatively charged already, i.e. it has a population of $n_{\text{n-SRH}}$.
$C_{\text{e,p-SRH}}$: an electron is captured into the positive SRH center. *It is a charge-recombination event.* It is only possible if the center is positively occupied already with population $p_{\text{p-SRH}}$.
$E_{\text{e,p-SRH}}$: an electron is emitted from the positive SRH center. It is possible if the center is neutral, which has a population of $N_{\text{p-SRH}} - p_{\text{p-SRH}}$.

$C_{\text{h,n-SRH}}$: a hole is captured into the negative SRH center. *It is a charge-recombination event.* It is possible if the trap state is negatively charged, i.e. it has a population of $n_{\text{n-SRH}}$.
$E_{\text{h,n-SRH}}$: a hole is emitted from the negative SRH center. It is equivalent to the excitation of an electron from the valence band into the trap level. It is only possible if the center is neutral, i.e. it has a population of $\left(N_{\text{n-SRH}} - n_{\text{n-SRH}}\right)$.
$C_{\text{h,p-SRH}}$: a hole is captured in the positive SRH center (is is equivalent to the emission of an electron from the neutral trap level). It is only possible if this trap center is originally neutral, i.e. it has a population of $\left(N_{\text{p-SRH}} - p_{\text{p-SRH}}\right)$.

$E_{\text{h,p-SRH}}$: a hole is emitted from the positive SRH center (is is equivalent to the activation of an electron from the VB to the trap level). It is only possible if this trap center is originally positively charged, i.e. it has a population of $p_{\text{p-SRH}}$.

Note the sign changes between the equations: the term which is positive for the CB (red), appears with a negative sign for the negative trap level but with the same positive sign for the positive trap level (blue). Similarly, the terms which are positive for the VB (green) appear with the same sign for the negative trap level but with the opposite sign for the positive trap level (black). This not only follows the logic of the emission and capture events but also guarantees the charge neutrality of the systems which in this case is equivalent to: $n + n_{\text{n-SRH}} = p + p_{\text{p-SRH}}$.

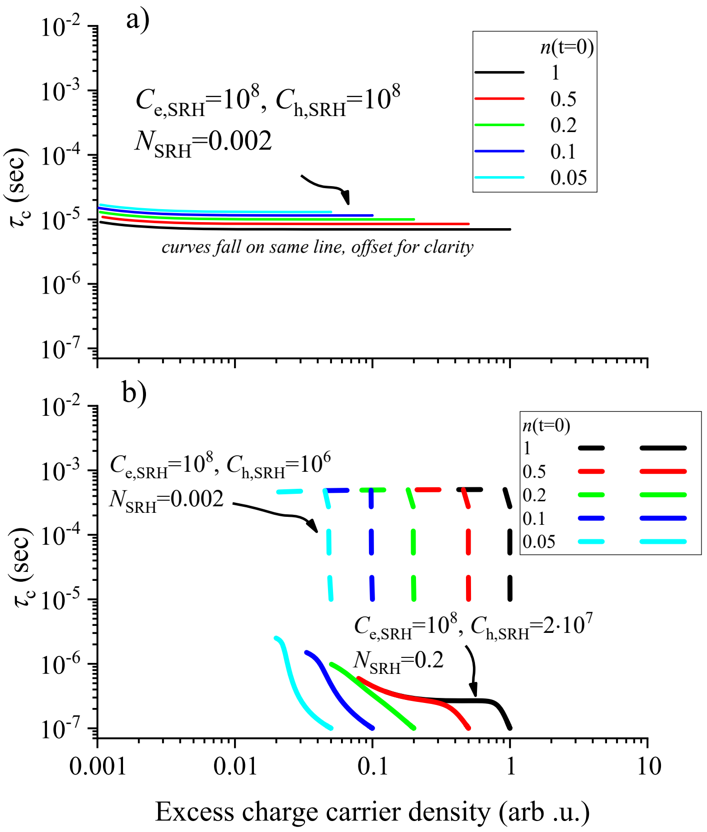

FIG. S1. Calculated charge-carrier lifetimes with the Shockley-Read-Hall model as discussed in the text. a) Shows the conventional SRH result when the electron and hole-capture rates are equal. The data fall on the same curve but are offset vertically for better visibility. b) Shows the same data as in the main text, which simulates well the experimentally observed result.

In FIG. S1. we show the simulation results with our SRH model for a conventional SRH result (a) and the same simulation results as in the main text (b).

In FIG. S2. we show the charge-carrier lifetime data for a fixed laser excitation energy for a number of different temperatures. Note that in the main text, the data is normalized to the same starting voltage value due to the temperature dependent photoconductivity of the sample. In contrast, FIG. S2. shows the raw data.

1. Shockley, W.; Read, W.T. Statistics of the Recombinations of Holes and Electrons. *Phys. Rev.* **1952**, *87*, 835–842.
2. Hall, R.N. Electron-Hole Recombination in Germanium. *Phys. Rev.* **1952**, *87*, 387–387.

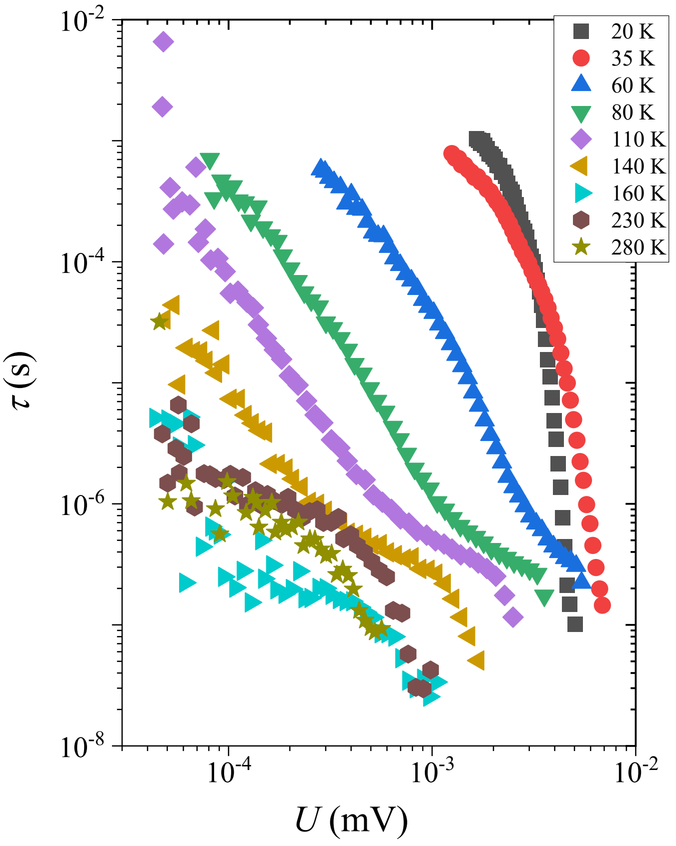

FIG. S2. Temperature dependence of the charge-carrier lifetime at different temperatures for a fixed initial laser excitation energy of 170 μJ/cm^2.

3. Hall, R. Recombination processes in semiconductors. *Proc. Inst. Electr. Eng., Part B* **1959**, *106*, 923–931(8).
